# Perturbing phosphoinositide homeostasis oppositely affects vascular differentiation in *Arabidopsis thaliana* roots

**DOI:** 10.1242/dev.155788

**Published:** 2017-10-01

**Authors:** Bojan Gujas, Tiago M. D. Cruz, Elizabeth Kastanaki, Joop E. M. Vermeer, Teun Munnik, Antia Rodriguez-Villalon

**Affiliations:** 1 Department of Biology, Swiss Federal Institute of Technology (ETH) Zurich, CH-8092, Zurich, Switzerland; 2 Department of Plant and Microbial Biology, University of Zurich, CH-8008, Zurich, Switzerland; 3 Section Plant Cell Biology, Swammerdam Institute for Life Sciences, University of Amsterdam, 1090 GE, Amsterdam, The Netherlands

**Keywords:** Phloem, Xylem, Vacuole, Intracellular trafficking, BFA, Programmed cell death

## Abstract

The plant vascular network consists of specialized phloem and xylem elements that undergo two distinct morphogenetic developmental programs to become transport-functional units. Whereas vacuolar rupture is a determinant step in protoxylem differentiation, protophloem elements never form a big central vacuole. Here, we show that a genetic disturbance of phosphatidylinositol 4,5-bis-phosphate [PtdIns(4,5)P_2_] homeostasis rewires cell trafficking towards the vacuole in *Arabidopsis thaliana* roots. Consequently, an enhanced phosphoinositide-mediated vacuolar biogenesis correlates with premature programmed cell death (PCD) and secondary cell wall elaboration in xylem cells. By contrast, vacuolar fusion events in protophloem cells trigger the abnormal formation of big vacuoles, preventing cell clearance and tissue functionality. Removal of the inositol 5′ phosphatase COTYLEDON VASCULAR PATTERN 2 from the plasma membrane (PM) by brefeldin A (BFA) treatment increases PtdIns(4,5)P_2_ content at the PM and disrupts protophloem continuity. Conversely, BFA application abolishes vacuolar fusion events in xylem tissue without preventing PCD, suggesting the existence of additional PtdIns(4,5)P_2_-dependent cell death mechanisms. Overall, our data indicate that tight PM phosphoinositide homeostasis is required to modulate intracellular trafficking contributing to oppositely regulate vascular differentiation.

## Abstract (precis)

**Summary:** Disturbance of the optimal ratio of PtdIns(4,5)P_2_ and PtdIns4P has opposing effects on vascular differentiation due to the distinct role of vacuolar biogenesis in their differentiation programs.

## INTRODUCTION

In *Arabidopsis thaliana*, as well as in other higher plants, the vascular system constitutes an inter-organ communication network that enables plants to respond to developmental and environmental stimuli (Lucas et al., 2013; Ruiz-Medrano et al., 2001). Whereas xylem tissues transport water and nutrients absorbed by the root to the above-ground organs, phloem sieve elements deliver photoassimilates and signaling molecules throughout the whole plant body (De Rybel et al., 2016; Lucas et al., 2013). Vasculature is symmetrically arranged within the root meristem, an organogenic center required to maintain post-embryonic organ growth, with a central xylem axis that is flanked by two phloem poles opposite to each other (Dolan et al., 1993; Lucas et al., 2013; Rodriguez-Villalon et al., 2014). The early initial phloem, termed protophloem, differentiates within the root meristem and elongates afterwards (Depuydt et al., 2013; Furuta et al., 2014; Scacchi et al., 2010; Truernit et al., 2012). Xylem differentiation occurs in the maturation zone of the root, and includes programmed cell death (PCD) and autolysis as part of its differentiation program. In particular, xylem maturation starts with the deposition of secondary cell wall (SCW) followed by vacuolar swelling (Escamez and Tuominen, 2014; Schuetz et al., 2013). As the tonoplast breaks down, vacuolar autolytic content is released promoting organelle disintegration and cytosol clearing, concomitant with the ongoing lignification (Bollhoner et al., 2012; Kuriyama, 1999; Pesquet et al., 2013). At the end of this process, primary cell walls will be partially degraded and perforation plates will be formed, conferring the characteristic helical pattern observed in tracheary elements (TEs) (Nakashima et al., 2000; Schuetz et al., 2013). By contrast, protophloem differentiation starts with primary cell wall thickening that occurs only upon emergence of small vacuole-like vesicles, which are proposed to emerge from the endoplasmic reticulum (ER) and travel to the plasma membrane (PM) (Furuta et al., 2014; Truernit et al., 2012; Wu and Zheng, 2003). As differentiation proceeds, the nucleus shrinks and the cytosol dilutes, eventually culminating in the disintegration of the majority of the organelles (Furuta et al., 2014). Notably, the conversion of vascular cells in organelle-depleted elements is a tightly regulated morphogenetic process, even if the underlying molecular mechanisms remain poorly understood (Fukuda, 2000; Furuta et al., 2014).

**Fig. 1.**
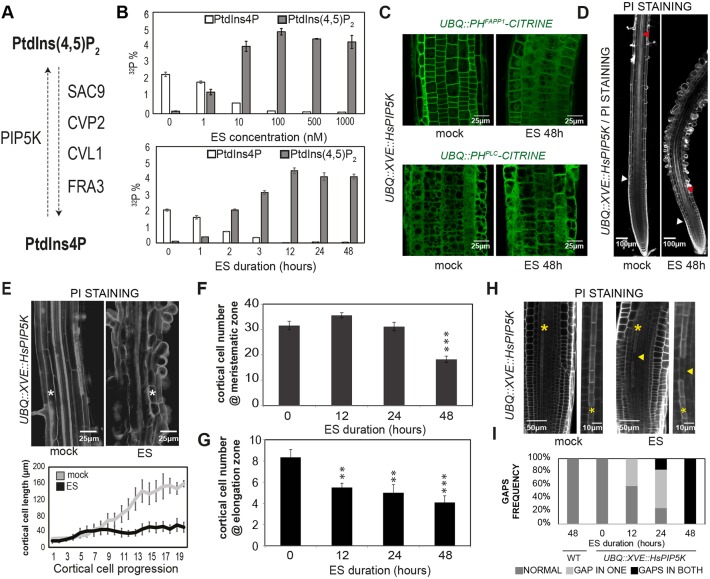
**An estradiol (ES)-inducible genetic tool to increase PtdIns(4,5)P_2_ levels.** (A) Schematic of phosphatidylinositol 4-phosphate (PtdIns4P) conversion into phosphatidylinositol 4,5-bis-phosphate [PtdIns(4,5)P_2_]. *CVL1*, *CVP2 LIKE 1*; *CVP2*, *COTYLEDON VASCULAR PATTERN 2*; *FRA3*, *FRAGILE FIBER 3*; PIP5K, PI4P 5′KINASE; *SAC9*, *SUPPRESSOR OF ACTIN9*. (B) Quantification of normalized PtdInsP and PtdInsP_2_ levels upon increasing estradiol (ES) concentrations and different incubation times in *UBQ::XVE::HsPIP5K* line. (C) Subcellular distribution of the PtdIns4P biosensor *UBQ::PH^FAPP1^-CITRINE* (top) and PtdIns(4,5)P_2_ biosensor *UBQ::PH^PLC^-CITRINE* (bottom) upon 48 h 0.5 μM ES treatment. (D) Root phenotype upon 48 h 0.5 μM ES-mediated *HsPIP5K* induction. White triangle marks the end of the meristematic zone whereas red triangle marks the appearance of first differentiated protoxylem strand. (E) Estradiol effect on cell growth. White asterisks mark cortical cells. On the lower panel, quantification of cortical cell length from transition zone onwards in mock- and ES-treated *UBQ::XVE::HsPIP5K* roots is represented (*n*=8). (F,G) Time-course analysis of meristematic activity (F) and elongation zone (G) in *UBQ::XVE::HsPIP5K* roots upon 0.5 μM ES-mediated *HsPIP5K* induction. (H) Undifferentiated protophloem gap cells marked by yellow triangle in PI-stained roots treated for 48 h with 0.5 µM ES. Yellow asterisks mark protophloem strands. (I) Quantification of gap presence in one or two strands in 5-day-old roots upon 0.5 μM ES-mediated *HsPIP5K* induction (*n*=12). Representative images (*n*>15 roots analyzed) are displayed for each treatment. Data represent mean±s.e.m. **P≤0.001, ***P≤0.0001 (Student's *t*-test between mock- and ES-treated roots).

Phosphatidylinositol 4,5-bis-phosphate [PtdIns(4,5)P_2_] is a minor constituent of membranes that belongs to a large family of signaling compounds called phosphoinositides, which are asymmetrically distributed among the diverse organelle membranes (Simon et al., 2014; Vermeer and Munnik, 2013). Such gradients, widely used by eukaryotic cells to directionally control vesicle trafficking within the cell (Heilmann and Heilmann, 2015; Ischebeck et al., 2013), are generated and maintained by the activity of phosphoinositide phosphatases and kinases (Fig. 1A) (Munnik and Nielsen, 2011). Besides its function as a membrane signaling compound, PtdIns(4,5)P_2_ plays a crucial role in controlling cell polarity and plant development (Munnik and Nielsen, 2011; Tejos et al., 2014). As such, the activity of two related phosphatidylinositol 4-phosphate (PtdIns4P) 5-kinases (Fig. 1A), PIP5K1 and PIP5K2, are required to regulate the clathrin-mediated endocytosis of two auxin efflux PIN-FORMED (PIN) transporters, PIN1 and PIN2, thus controlling their polar localization at the PM (Ischebeck et al., 2013; Tejos et al., 2014). Moreover, protophloem PtdIns(4,5)P_2_ pools are tightly controlled by the activity of two phloem-specific phosphatases, COTYLEDON VASCULAR PATTERN 2 (CVP2, At5PTase6) and its partially redundant homolog CVP2 LIKE 1 (CVL1, At5PTase7) (Fig. 1A, Fig. S4A) (Carland and Nelson, 2009; Ercetin et al., 2008; Gillaspy, 2011; Rodriguez-Villalon, 2015). These enzymes are required to ensure an optimal progression of the phloem differentiation program as revealed by the appearance of undifferentiated cells, the so-called gap cells, in *cvp2 cvl1* root protophloem strands (Fig. 4A) (Rodriguez-Villalon et al., 2015). Interestingly, both PtdIns4P and PtdIns(4,5)P_2_ stimulate the activity of VAN3 (also known as SCARFACE), an ARF-GAP protein involved in regulating membrane trafficking in the post-Golgi transport pathway (Naramoto et al., 2009). Yet, how PM PtdIns(4,5)P_2_ pools orchestrate the subcellular rearrangement associated with vascular differentiation remains poorly understood. Here, we show how a skewed PtdIns(4,5)P_2_/PtdIns4P ratio redirects vesicle trafficking towards the vacuole and, in turn, promotes vacuolar fusion events. Remarkably, this phenomenon modulates cell elongation and has opposing effects on xylem and phloem differentiation programs. On the one hand, enhanced vacuolar biogenesis correlates with a premature PCD execution and SCW building in xylem tissues. On the other hand, the abnormal formation of big vacuolar structures in mature protophloem cells accounts for the defective tissue functionality observed in a genetic background with impaired PtdIns(4,5)P_2_/PtdIns4P homeostasis (Rodriguez-Villalon et al., 2015). Moreover, pharmacological interference with the intracellular recycling of CVP2 from *trans*-Golgi network (TGN) to the PM by brefeldin A (BFA) mimics the *cvp2 cvl1* vascular phenotype in terms of atypical big vacuole formation. By contrast, BFA treatment prevents vacuole swelling in xylem cells, although it does not prevent PCD occurrence, implying the existence of a vacuole-uncoupled PtdIns(4,5)P_2_ regulatory mechanism. Our data suggest that tissue-specific PtdIns(4,5)P_2_ turnover meets the requirements to generate a dual mechanism allowing the cell to regulate differentiation programs antagonistically in vascular cells.

## RESULTS

### Balanced PtdIns(4,5)P_2_ homeostasis is essential to sustain root cell elongation and modulate cell differentiation

Optimal vascular formation requires a tight balance between PtdIns4P and PtdIns(4,5)P_2_ (Carland and Nelson, 2004; Rodriguez-Villalon, 2015). The different morphogenetic programs shaping phloem and xylem cells as conducting elements prompted us to compare phosphoinositide function in xylem and phloem differentiation. Whereas addressing this question in phloem tissue is possible owing to the existence of tissue-specific loss-of-function *5′-ptases*, the broad expression of 5PTases predicted to target xylem tissues such as *FRAGILE FIBER 3* (*FRA3*) and *SUPPRESSOR OF ACTIN 9* (*SAC9*) has hampered the analysis of phosphoinositides' role in xylem tissues (Fig. 1A) (Brady et al., 2007; Zhong et al., 2004). Therefore, we sought to establish an inducible genetic system to increase PtdIns(4,5)P_2_ levels. Constitutive heterologous expression of a human PIP5KIα (*HsPIP5K*) in *Nicotiana tabacum* has been reported to increase PtdIns(4,5)P_2_ 100-fold, mainly at the PM (Im et al., 2007, 2014). To prevent undesired developmental defects, we introduced *HsPIP5K* under the control of an estradiol-inducible cassette (*XVE*) driven by *UBIQUITIN 10* (*UBQ*) promoter and assessed its metabolic functionality. Measurements of phosphoinositide content by ^32^Pi (radioactively labelled inorganic phosphate; ^32^P). labeling and thin-layer chromatography (TLC) revealed a positive estradiol dosage- and time-dependent correlation between phosphatidylinositol monophosphate (PtdInsP) consumption and phosphatidylinositol bisphosphate (PtdInsP_2_) production at the PM, as inferred by the subcellular localization of HsPIP5K protein tagged with GFP (Fig. 1B, Fig. S1A). PtdIns4P consumption can be verified by use of its biosensor *UBQ::PH^FAPP1^-CITRINE*, which in mock conditions mainly resides at the PM, whereas such localization is abolished upon *HsPIP5K* induction (Fig. 1C) (Vermeer et al., 2009). Strangely, however, high PtdIns(4,5)P_2_ production when inducing *HsPIP5K* expression was not revealed by *UBQ::PH^PLC^-CITRINE* cytosolic localization (Fig. 1C) (van Leeuwen et al., 2007). The latter may indicate that the PtdIns(4,5)P_2_ formed is not accessible to the cytosolic fluorescent probe, for example because the lipid is mainly bound to endogenous PtdIns(4,5)P_2_ targets, which have a higher affinity than the PtdIns(4,5)P_2_-binding site of the biosensor. What is clear from the ^32^Pi-labeling, however, is that *HsPIP5K* induction causes a massive change in PtdIns(4,5)P_2_ and PtdIns4P ratio. Although such phosphoinositide accumulation has never been observed in wild-type *Arabidopsis* seedlings, some developmental effects observed in *HsPIP5K*-induced roots matched previous reports (Ischebeck et al., 2013; Rodriguez-Villalon et al., 2015). For instance, perturbation of PtdIns(4,5)P_2_/PtdIns4P ratio upon *HsPIP5K* induction for 48 h caused a major arrest of post-embryonic root growth (Fig. S1B). The origin of this phenotype could be traced to reduced meristematic activity and hampered cell elongation rate (Fig. 1D-F), as revealed by the quantification of root cortical cell number and length when inducing *HsPIP5K* expression (Fig. 1E,F). Furthermore, a pleiotropic effect caused by *HsPIP5K* induction involved a series of premature differentiation events related to epidermis, endodermis and xylem cells. In particular, we observed that elevated PtdIns(4,5)P_2_ levels do not only severely affect root hair initiation and elongation, consistent with previous reports (Fig. S1C) (Im et al., 2014; Ischebeck et al., 2013), but also stimulate endodermis differentiation as manifested by the early expression of *CASPARIAN STRIP MEMBRANE DOMAIN PROTEIN 1* (*CASP1*) (Fig. S1D) (Roppolo et al., 2011). Likewise, *HsPIP5K* induction shifted the expression of the xylem maturation marker *S18::GFP* closer to the root meristem in comparison with wild-type plants, a trait that closely correlates with the appearance of the first protoxylem differentiating cell when increasing PtdIns(4,5)P_2_ levels (Fig. 1D, Fig. S1E). To avoid systemic effects due to prolonged *HsPIP5K* induction, we next assessed various root traits in a time-course experiment. The first developmental outcome was observed 12 h after *HsPIP5K* induction and was reflected in a decreased root elongation zone, as quantified by counting cortical cell number from the transition zone until the first hallmark of root differentiation zone, i.e. the appearance of an SCW in xylem strands (Fig. 1G). Interestingly, a reduction of meristematic activity can only be detected after 48 h (Fig. 1F) and is preceded by the appearance of undifferentiated gap cells – 12 h after *HsPIP5K* induction – in at least one protophloem strand (Fig. 1H,I). The latter was consistent with previous reports showing that a tight PtdIns(4,5)P_2_/PtdIns4P ratio is required to ensure continuity of protophloem strands, and, in turn, optimal root meristematic activity (Rodriguez-Villalon et al., 2015). Overall, our results suggest that increased PtdIns(4,5)P_2_ levels limit cell growth and modulate the differentiation programs of root vascular tissues in an opposing manner.

### Increased PtdIns(4,5)P_2_ turnover stimulates intracellular trafficking towards the vacuole

**Fig. 2.**
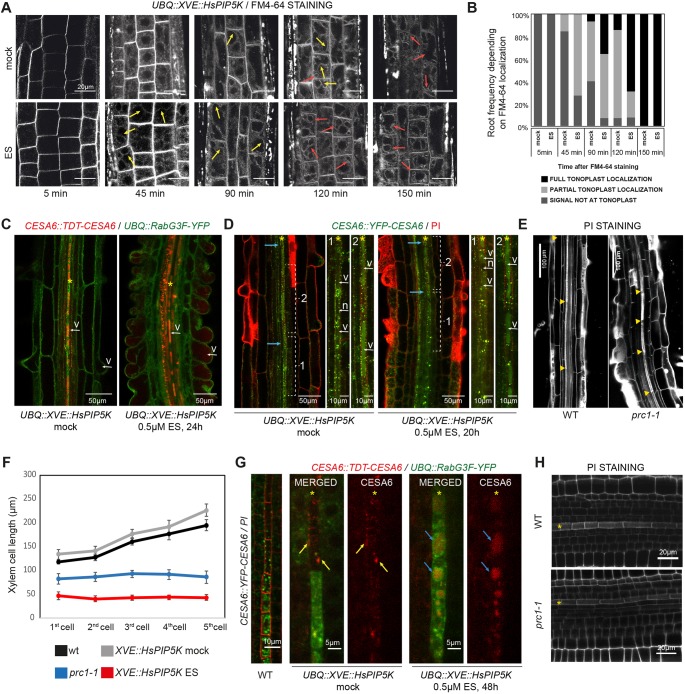
**High PtdIns(4,5)P_2_ levels enhanced intracellular trafficking towards the vacuole.** (A,B) FM4-64 uptake in *HsPIP5K*-induced seedlings at the specified times. Note that after 45 min FM4-64 is partially localized (yellow arrows) in the tonoplast in an elevated PtdIns(4,5)P_2_ background. Red arrows indicate full FM4-64 localization at the tonoplast. (B) Quantification of total or partial localization of FM4-64 signal at the tonoplast. Frequency of number of roots harboring the labeled trait [*n*=4-5 roots per experiment (3 independent experiments)/time point]. (C) tdTOMATO-CESA6 accumulation in epidermal (root hair) cells upon *HsPIP5K* induction. v, vacuole. (D) Analysis of *CESA6::YFP-CESA6* localization shift from intracellular compartments to the vacuole in xylem strands. Blue arrows indicate the first cell building SCW. Note the difference in CESA6 content between vacuoles (v) in cells before (1) and after (2) SCW. Also note that in ES-induced *HsPIP5K* line the xylem cell before SCW formation (1) has homogeneous YFP-CESA6 in the vacuole. YFP-CESA6 signal is never present in the nucleus (n). (E) Examination of protoxylem cell length in PI-stained roots of Col and *cesa6* mutant (*prc1-1*). Yellow triangles mark cell ends. (F) Quantification of the cell length of the first five protoxylem cells with formed SCW in the indicated genotypes. Roots were subjected to 5 µM estradiol treatment for 48 h before analysis. Data represent mean±s.e.m. (G) Protophloem YFP-CESA6 localization in PI-stained roots and colocalization of *tdTOMATO-CESA6* with the vacuolar/late endosome marker RabG3F-YFP in root protophloem cells upon *HsPIP5K* induction for 48 h with 0.5 µM ES. Yellow arrows mark PM localization of CESA6 whereas blue arrows mark vacuolar localization of the protein. (H) Analysis of protophloem strands of Col and *prc1-*1 roots stained with PI. Representative images (*n*≥12 roots analyzed) are displayed for each treatment/genotype and asterisks mark vascular strand. WT, wild type.

To further substantiate the hypothesis that tight PtdIns(4,5)P_2_/PtdIns(4)P homeostasis is required to reorganize vascular cell content during the process of cell differentiation, we next analyzed subcellular trafficking dynamics by confocal live imaging. To do so, we took advantage of the widely used cell trafficking dye FM4-64 (Rigal et al., 2015). In particular, FM4-64 stains PM and over time is distributed throughout the vesicular network from PM to the vacuole by vesicular integration (Fig. S1F) (Rigal et al., 2015). Comparison of wild-type and *HsPIP5K*-induced roots showed faster arrival of FM4-64 to the tonoplast in conditions with higher PtdIns(4,5)P_2_ levels (Fig. 2A,B, Fig. S1F). The latter implies that faster PtdIns(4,5)P_2_ turnover stimulates trafficking of cell vesicles towards the vacuole in *Arabidopsis* epidermal cells. Unfortunately, the poor penetrance of FM4-64 to the vascular cylinder limits its use to the ground tissues. Thus, we decided next to analyze *CELLULOSE SYNTHASE 6* (CESA6), a subunit responsible for primary cell wall formation and for which abundance at the PM depends on membrane trafficking (Fagard et al., 2000; Fujimoto et al., 2015; Zhang et al., 2016). To test whether boosting PtdIns(4,5)P_2_ levels would also rewire intracellular trafficking of CESA6 towards the vacuole, we monitored tdTOMATO-CESA6 behavior together with the late endosome/vacuole marker RabG3F-YFP (Geldner et al., 2009) by confocal microscopy.

Interestingly, upon *HsPIP5K* induction, CESA6 accumulated in vacuoles of epidermal cells that started root hair budding, a phenomenon never observed in wild-type seedlings, supporting our FM4-64 findings (Fig. 2A,C). However, CESA6 can be found in vacuoles of differentiating xylem cells even in mock conditions (Fig. 2C, asterisk). In particular, a sharp change in YFP-CESA6 localization towards the vacuole was observed concomitant with SCW formation (Fig. 2D). Such drastic change in the subcellular localization of CESA6 occurred closer to the root meristem in *HsPIP5K*-induced root, perfectly correlating with a premature xylem SCW elaboration observed in these seedlings (Fig. 2D). Because a vacuolar YFP-CESA6 localization in *HsPIP5K*-induced roots can be often observed in one or two cells without visible SCW morphology, it seems plausible that primary cellulose synthase subunit loading into the vacuole precedes secondary cell wall formation. Nevertheless, premature vacuolar sequestration of CESA6 might explain reduced xylem cell elongation in roots with elevated PtdIns(4,5)P_2_ (Fig. 2D), as mutants with impaired cell wall formation cannot cope with an optimal vacuolar-driven cellular growth (Fagard et al., 2000). In agreement with this notion, examination of protoxylem cell size in *prc1-1* mutants with deficient CESA6 activity revealed a reduced cell elongation rate compared with wild-type xylem strands (Fig. 2E,F). Such a phenotype is reminiscent of the cellular growth observed upon perturbing PtdIns(4,5)P_2_ homeostasis (Fig. 2F), even if at this stage we cannot exclude additional factors responsible for the reduced cell length. Likewise, we analyzed CESA6 localization in protophloem differentiating cells. Whereas YFP-CESA6 was localized in internal compartments in undifferentiated protophloem elements, YFP-CESA6 could only be detected at the PM in mature cells (Fig. 2G). Unlike in mock-treated roots (Fig. 2G), *HsPIP5K*-induction triggered the formation of big vacuoles and sequestration of CESA6 into them (Fig. 2G). Such a phenotype was only observed in mature protophloem cells exhibiting thick cell walls, implying that this feature does not account for the defective gap phenotype observed in *HsPIP5K*-induced roots (Fig. 2G, Fig. 1H). Moreover, morphological examination of *prc1-1* roots did not reveal disrupted protophloem continuity (Fig. 2H), suggesting that even if CESA6 contributes to the elaboration of the primary cell wall in protophloem differentiating cells, it is not essential in this process. Altogether, our results suggest that high PtdIns(4,5)P_2_ turnover stimulates cell trafficking towards the vacuole in epidermal, xylem and phloem cells, and alters the subcellular distribution of at least one CESA subunit from PM to the vacuolar cell compartment.

### Increased PtdIns(4,5)P_2_ turnover stimulates protoxylem differentiation

**Fig. 3.**
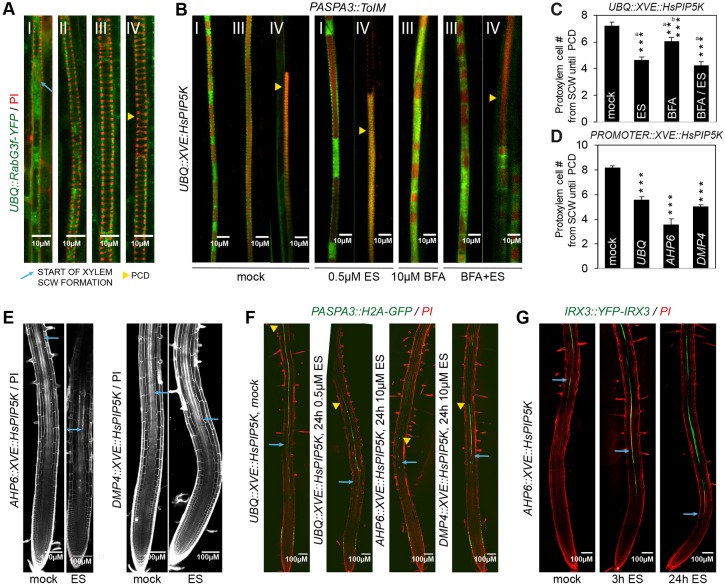
**Increased PI(4,5)P_2_ concentration affects vacuolar biogenesis and PCD in differentiating xylem cells.** (A) Visualization of differentiating xylem cells in a 5-day-old PI-stained root by monitoring the vacuolar marker *UBQ::RabG3f-YFP*. Stage I refers to the first cell where SCW could be detected, whereas cells displaying several vacuoles after SCW formation are marked as stage II. The cell occupied by only one big vacuolar compartment just before PCD execution is labeled as stage III. Stage IV refers to cells in which the tonoplast is breaking down or cannot be detected any longer due to its rupture. (B) Vacuolar morphology in protoxylem cells of 24 h 0.5 µM ES-treated roots that were subjected to 10 µM BFA treatment. Yellow triangle marks tonoplast breakdown as assessed by the colocalization of red (vacuolar) and green (cytosol) signals. Note that upon ES treatment a big vacuole occupies the majority of the cellular space in stage I xylem cells. (C,D) Effect of increased PtdIns(4,5)P_2_ levels and BFA treatment on PCD progression. Quantification of cells exhibiting *PASPA3::H2A-GFP* expression as a marker for nuclei presence from SCW formation until PCD upon the indicated treatments and in the indicated transgenic lines. Data represent mean±s.e.m. **P≤0.001, ***P≤0.0001 [Student's *t*-test between mock (a) and ES (b) treatment]. (E) Confocal microscopy analyses of *AHP6::XVE::HsPIP5K* and *DMP4::XVE::HsPIP5K* roots germinated on 10 µM ES. Blue arrows mark the appearance of protoxylem differentiated cells based on cell wall morphology. (F) Expression analysis of the PCD-associated *PASPA3* gene upon increasing PtdIns(4,5)P_2_ concentration in the indicated transgenic lines. Note that blue arrows mark the onset of SCW formation whereas the yellow triangle marks the last nucleated cell. (G) Confocal microscopy analyses of *IRX3::YFP-IRX3* in *AHP6::XVE::HsPIP5K*. *HsPIP5K* expression was induced by 10 µM ES for the indicated times. Blue arrows mark the appearance of protoxylem differentiated cells based on cell wall morphology.

Xylem maturation starts with the deposition of SCW followed by vacuolar swelling (Escamez and Tuominen, 2014; Schuetz et al., 2013). As optimal vacuolar formation depends on vesicle trafficking towards this compartment (Gao et al., 2009; Zhang et al., 2014), it is possible that PtdIns(4,5)P_2_ species stimulate xylem differentiation by modulating vacuolar biogenesis. Moreover, vacuolar fusion events precede tonoplast rupture when vacuolar autolytic content is released triggering organelle disintegration and cell clearing (Bollhoner et al., 2012; Kuriyama, 1999; Pesquet et al., 2013). Confocal microscopy examination of protoxylem strands stained with propidium iodide (PI) – a dye that mainly stains demethoxylated pectin content at the cell wall (Rounds et al., 2011) – revealed the appearance of the characteristic helical spiral before the occurrence of big vacuolar fusion events, as demonstrated by the analysis of the late endosome/vacuolar marker *RabG3f-YFP* (Fig. 3A). Moreover, an increase in vacuolar size such that it occupies the majority of the cell, perfectly correlated with PCD, as inferred by the absence of the tonoplast (Fig. 3A). To test whether enhanced trafficking towards the vacuole driven by elevated PtdIns(4,5)P_2_ levels would accelerate the final steps of xylem differentiation, we took advantage of the recently described ToIM genetic fluorescent tool, which decorates vacuoles with RFP whereas the cytosol becomes visible by the presence of GFP (Fendrych et al., 2014). In particular, we crossed *UBQ::XVE::HsPIP5K* with the ToIM marker driven by the *PASPA3* promotor, an aspartic protease specifically expressed in tissues undergoing PCD such as lateral root cap and xylem (Fendrych et al., 2014). This genetic tool enabled us to easily distinguish xylem from other vascular cylinder tissues and confirm that increased PtdIns(4,5)P_2_ levels positively correlate with vacuolar size expansion, a phenomenon that can be observed in developmentally younger xylem cells (Fig. 3B). Unlike in mock strands, in which a gradient of vacuolar fusion events was observed, only big vacuoles were detected in protoxylem cells of *HsPIP5K*-induced roots (Fig. 3B). Furthermore, tonoplast rupture occurrence in protoxylem cells upon *HsPIP5K* induction as revealed by colocalization of vacuolar (red) and cytosolic (green) signals (yellow triangle) (Fig. 3B) seemed to happen prematurely in comparison with mock roots. In order to corroborate this finding, we followed nuclei disintegration along a protoxylem strand of *PASPA3::H2A-GFP* roots (Fendrych et al., 2014). Whereas in mock conditions *PASPA3::H2A-GFP* expression starts before the onset of SCW formation and extends until the nucleus disintegrates (as confirmed by the ToIM marker line under the same promotor), *HsPIP5K* induction led to a significant decrease of cells labeled with *H2A-GFP* signal, suggesting faster PCD occurrence (Fig. 3C). These observations indicated a positive correlation between enhanced vacuolar biogenesis and the premature xylem differentiation observed upon increasing PtdIns(4,5)P_2_ levels. Next, to test causality of the vacuolar size and PCD, we chemically blocked vesicle sorting from the TGN to lytic vacuole by brefeldin A (BFA), a compound that targets ADP-ribosylation GTP-exchange factors (ARF-GEFs) and thus blocks intracellular trafficking at the level of the TGN (Dettmer et al., 2006; Geldner, 2004; Kleine-Vehn et al., 2008; Robineau et al., 2000; Tse et al., 2007). Interestingly, the enhanced vacuolar biogenesis observed upon *HsPIP5K* induction was abolished when roots were treated with 10 µM BFA for 24 h (Fig. 3B), demonstrating that an intact endomembrane system is required to promote PtdIns(4,5)P_2_-dependent vacuolar fusion events in xylem tissue. Interestingly, even though only small vacuoles could be observed in xylem strands upon BFA treatment, PCD was not prevented, and was still occurring faster in *HsPIP5K*-induced roots in comparison with mock (Fig. 3B,C). The latter observation suggested the existence of PtdIns(4,5)P_2_-dependent additional factors promoting xylem PCD (Fig. 3C). To understand better the spatiotemporal requirements of PtdIns(4,5)P_2_ turnover during xylem differentiation, we decided to express the *HsPIP5K* construct in protoxylem cells undergoing different developmental stages. To this aim, we took advantage of the protoxylem-specific expression of *ARABIDOPSIS HISTIDINE PHOSPHOTRANSFER PROTEIN 6* (*AHP6*) (Mahonen et al., 2006) and *DMP4*, a paralog of *DOMAIN OF UNKNOWN FUNCTION 679 MEMBRANE PROTEIN 2* (*DMP2*) (Olvera-Carrillo et al., 2015). Whereas *AHP6* starts to be expressed in protoxylem meristematic cells, *DMP4* targets protoxylem cells already undergoing SCW formation (Fig. S2A). Notably, the meristematic zone was not affected before 48 h of *HsPIP5K* induction even though a premature differentiated xylem strand could be observed in *AHP6::XVE::HsPIP5K* roots, but not in *DMP4::XVE::HsPIP5K*, in accordance with their expression patterns (Fig. 3E, Fig. S2B). Consistent with our previous observations for the *UBQ::XVE::HsPIP5K* line, an increase in xylem vacuolar biogenesis was detected in the *AHP6::XVE::HsPIP5K* line when monitoring the *PASAP3::ToIM* marker by confocal microscopy (Fig. S2C). Moreover, a protoxylem-specific enrichment of PtdIns(4,5)P_2_ confirmed faster triggering of PCD in in comparison with wild-type roots as revealed by the analysis of H2A-GFP signal driven by the *PASPA3* promotor (Fig. 3D,F). Similarly, confocal microscopy analysis of nucleus persistence in protoxylem strands of roots stained with 4′,6-diamidino-2-phenylindole (DAPI) after SCW lignin deposition revealed a significant decrease in number of nucleated cells upon *HsPIP5K* induction (Fig. S2D). Conversely, an increase of PtdIns(4,5)P_2_ levels in protoxylem meristematic cells trigger the expression of *CELLULOSE SYNTHASE A7* (*CESA7*) also known as *IRREGULAR XYLEM 3* (*IRX3*) – the main cellulose biosynthetic enzyme responsible for xylem SCW formation (Taylor et al., 1999) – closer to the root meristem (Fig. 3G). Furthermore, the characteristic pitted cell wall morphology of differentiated metaxylem cells was prematurely detected upon *HsPIP5K-*induction compared with wild-type plants (Fig. S2E), even if at this stage the causality of this phenomenon cannot be elucidated. Quantification of differentiated protoxylem cell number until detection of the first metaxylem mature element assessed by its characteristic cell morphology corroborated this notion (Fig. S2F). Overall, our results suggest that an increase in PtdIns(4,5)P_2_ levels modulates intracellular trafficking towards the vacuole associated with xylem differentiation, a phenomenon that can be blocked by BFA treatment. However, additional PtdIns(4,5)P_2_-dependent factors unrelated to vacuolar biogenesis seem to trigger the onset of xylem differentiation programs, stimulating SCW formation and PCD.

### Enhanced trafficking towards the vacuole prevents protophloem differentiation

**Fig. 4.**
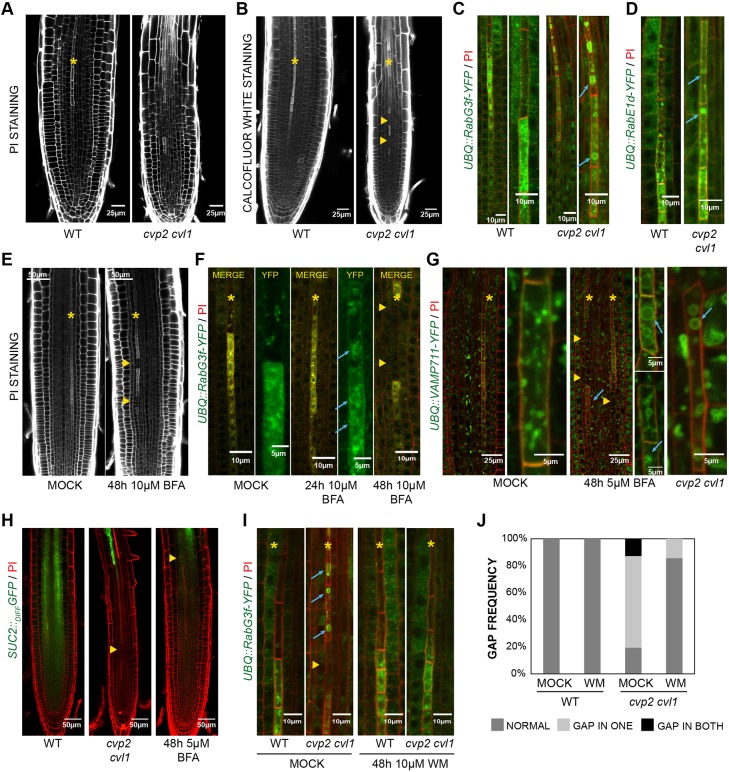
**Effect of impaired PI(4,5)P_2_ homeostasis on protophloem differentiation.** (A,B) Pectin accumulation (A) and cellulose deposition (B) in PI- and Calcofluor White-stained wild-type (WT) and *cvp2 cvl1* roots. (C,D) Visualization of late endosome and tonoplast (*UBQ::RabG3f-YFP*; C) and trafficking from the Golgi to PM (*UBQ::RabE1d-YFP*; D) were monitored by multi-photon confocal microscopy in 6-day-old WT and *cvp2 cvl1* roots stained with PI. Magnification of protophloem cells around enucleation point are displayed on the right (C). (E,F) Analysis of cell wall (E) and vacuolar morphology (F) upon 10 µM BFA treatment in PI-stained roots visualized by confocal microscopy. (G) BFA-triggered structures decorated with VAMP711-YFP in a protophloem differentiating cell upon BFA treatment in wild type and *cvp2 cvl1*. (H) Effect of an impaired protophloem differentiation program on root meristem unloading upon 5 µM BFA treatment. Confocal microscopy analysis of PI-stained roots harboring the meristem unloading marker *SUC2::GFPdiffusible*. (I) Restoration of normal vacuolar disintegration and cell wall thickening of undifferentiated protophloem cells in *cvp2 cvl1* upon 48 h of 10 µM wortmannin (WM) treatments. (J) Quantification of gap appearance in none, one or both protophloem strands in PI-stained roots visualized by confocal microscopy (*n*=12). Yellow asterisks mark protophloem strand, yellow triangles point at gap cells, blue arrows mark abnormal vacuoles in elongated protophloem cells. Representative images (*n*=15 roots analyzed) are displayed for each treatment/genotype.

To evaluate the impact of a PtdIns(4,5)P_2_-mediated trafficking towards the vacuole on protophloem differentiation, we decided to examine cell wall formation and vesicle trafficking towards the vacuole in a *cvp2 cvl1* background. Consistent with previous reports, a discontinuous progression of cell wall formation was observed when boosting PtdIns(4,5)P_2_ levels, as revealed by PI staining of *cvp2 cvl1* roots (Fig. 4A) (Rodriguez-Villalon et al., 2015). Likewise, Calcofluor White staining revealed an interrupted deposition of cellulose in protophloem differentiating cells (Fig. 4B). To confirm that the observed vascular phenotypes were due to a protophloem enrichment of PtdIns(4,5)P_2_, we next drove *XVE::HsPIP5K* expression under the protophloem-specific promoter *BARELY ANY MERISTEM 3* (*BAM3*) (Fig. S3) (Rodriguez-Villalon et al., 2014). Biochemical quantification of phosphoinositide levels in two independent *BAM3::XVE::HsPIP5K* transgenic lines revealed an increase of PtdIns(4,5)P_2_ levels upon *HsPIP5K* induction (Fig. S3A), a trend that perfectly correlates with the appearance of gap cells in the protophloem strand (Fig. S3B). Next, we decided to assess whether the phosphoinositide-dependent vascular phenotype could be traced to enhanced vacuolar biogenesis. Confocal microscopy analysis of *UBQ::RabG3f-YFP* demonstrated that, unlike in xylem cells, protophloem late endosomes never fuse into a centrally positioned vacuole and instead simply disintegrate (Fig. 4C). Interestingly, the formation of atypical big vacuole-like structures was detected in *cvp2 cvl1* and *BAM3::XVE::HsPIP5K* cells (Fig. 4C, Fig. S3C), implying that elevated PtdIns(4,5)P_2_ levels promote late endosome fusion events and vacuole formation in protophloem cells exhibiting thick cells walls. Furthermore, increased PtdIns(4,5)P_2_/PtdIns4P ratio in *cvp2 cvl1* double mutants also triggered aggregation of RabE1d-YFP – a protein that normally accumulates at the Golgi and translocates to the PM (Camacho et al., 2009) – in rounded structures (Fig. 4D). To evaluate the interdependence between a perturbed PtdIns(4,5)P_2_-mediated endomembrane system and the cell gap appearance in *cvp2 cvl1* protophloem cells, we decided next to suppress *cvp2 cvl1* vascular defects by BFA treatments. Surprisingly, incubation of wild-type seedlings for 48 h in 10 µM BFA led to the appearance of undifferentiated gap cells within the protophloem strands as revealed by their thin cell wall (Fig. 4E), mimicking *cvp2 cvl1* vascular phenotype (Fig. 4A). Moreover, time-course analysis of *UBQ::RabG3f-YFP* dynamics upon BFA treatment surprisingly showed after 24 h the formation of atypical large vesicular structures and aggregates, whereas cell wall morphology appeared intact (Fig. 4F). Together, these findings imply that the formation of vacuole-like structures in protophloem differentiating cells precedes the appearance of the thin-cell wall gap phenotype observed in *cvp2 cvl1* roots. These findings prompted us to analyze further the nature of such vesicle structures by using *UBQ::VAMP711-YFP*, a fluorescent-tagged chimeric protein specifically targeted to the tonoplast (Geldner et al., 2009), upon BFA treatment (Fig. 4G). Exhaustive examination of vesicle structures decorated with VAMP711-YFP revealed a progressive fusion of vesicles to the tonoplast, a subcellular scenario reminiscent of that observed in *cvp2 cvl1* roots (Fig. 4G). Together, these findings show that BFA treatment in protophloem cells has the opposite effect to that in epidermal or xylem cells, stimulating trafficking towards the vacuole. Moreover, it appears to be possible that the persistence of big vacuolar structures in BFA-treated wild-type seedlings in the transition and elongation zone of the root could cause defective tissue functionality as in *cvp2 cvl1* roots (Fig. 4H) (Rodriguez-Villalon et al., 2015). To corroborate this hypothesis, we next analyzed root meristem unloading of free fluorescent GFP reporter protein under the control of the companion cell-specific *SUC2* promoter in BFA-treated plants. Notably, GFP unloading was strongly reduced upon BFA treatment in comparison with mock-treated plants (Fig. 4H). Altogether, these observations denote that a fine-tuned phosphoinositide balance is required to prevent intracellular trafficking towards the vacuole, which in turn hampers protophloem cell clearance. Indeed, this notion was confirmed by rescuing *cvp2 cvl1* subcellular defects upon treatment with wortmannin (WM) (Fig. 4I), a widely used pharmaceutical compound that targets phosphatidylinositol 3-kinase and PtdIns4P kinases in a dose-dependent manner leading to the inhibition of protein vacuolar sorting. Remarkably, the exogenous application of 10 µM WM for 48 h not only led to the disappearance of the atypical rounded vesicles observed in *cvp2 cvl1* mature elements but it also restored cell wall morphology similar to wild type, whereas no visible effect could be observed in mock conditions (Fig. 4I,J). Likewise, the gap phenotype observed in *HsPIP5K*-induced protophloem strands was partially corrected to a wild-type situation in *BAM3::XVE::HsPIP5K* roots (Fig. S3D,E), implying that the PtdInsP_2_-dependent appearance of atypical big vacuoles might be coupled with flanking cell incapability to differentiate.

### CVP2 recycling is essential for tight regulation of PtdIns(4,5)P_2_ turnover at protophloem PM

**Fig. 5.**
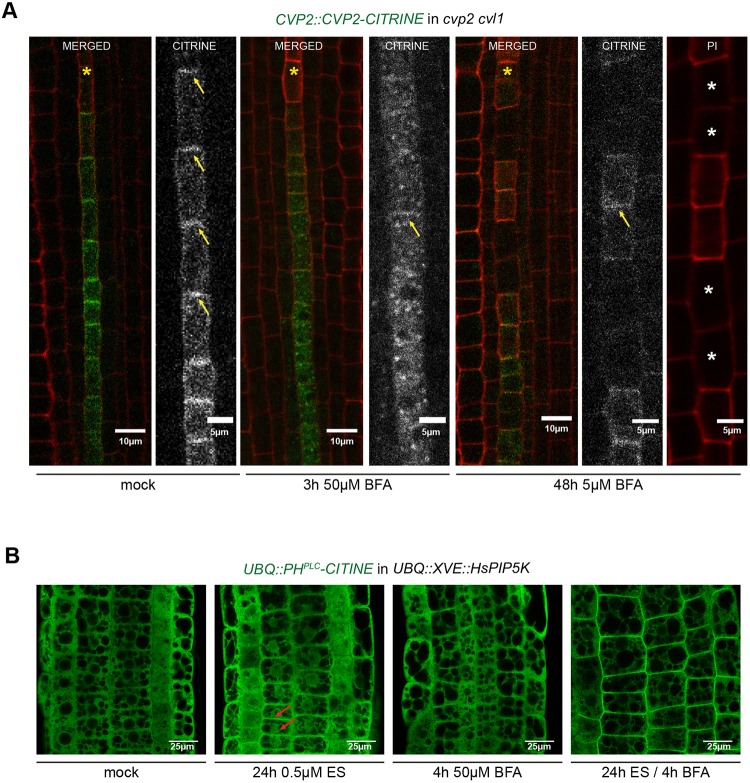
**Requirement of CVP2 localization at the PM to regulate protophloem PtdIns(4,5)P_2_ turnover.** (A) Analysis by multi-photon confocal microscopy of CVP2 subcellular localization and recycling in protophloem cells of PI-stained roots after treatment with BFA for the specified times. Arrows mark CVP2 PM localization. Yellow asterisks mark protophloem strands whereas white asterisks mark gap cells. (B) Analysis of PtdIns(4,5)P_2_ biosensor distribution in a *UBQ::XVE::HsPIP5K* epidermal root cell upon BFA treatments. Red arrows mark weak PM localization.

Because BFA exogenous application mimics the protophloem *cvp2 cvl1* phenotype, we next asked whether protophloem-specific phosphatases subjected to intracellular recycling are essential for controlling PtdIns(4,5)P_2_ turnover at the PM. To do so, we decided to examine by live imaging the localization of the protophloem-specific 5PTase CVP2 (Fig. S4A) upon BFA treatments. Firstly, analysis of *cvp2 cvl1* roots harboring a *CVP2::CVP2-CITRINE* construct revealed a complete root morphological rescue, as single *cvl1* mutant does not exhibit any detectable root phenotype (Rodriguez-Villalon et al., 2015). Next, we decided to analyze CVP2 localization by using multi-photon confocal microscopy. Interestingly, in protophloem root cells that are about to enter into differentiation programs, CVP2 accumulates polarly at the PM (Fig. 5A, Fig. S4A), although it can be also detected in certain intracellular compartments. Thus, it appears plausible that CVP2 PM localization is regulated by recycling mechanisms that are, in turn, responsible for the regulation of PtdIns(4,5)P_2_ turnover. To test this hypothesis, *CVP2::CVP2-CITRINE* roots were subjected to 3 h of 50 µM BFA. As expected, accumulation of *CVP2* in BFA-compartments (Fig. 5A) inversely correlates with its disappearance from the PM. Furthermore, prolonged BFA treatment for 48 h resulted in the appearance of gap cells in protophloem strands, resembling the *cvp2 cvl1* phenotype. Interestingly, in gap cells CVP2 was absent from PM, but the PM localization of CVP2 can still be detected in the cells exhibiting a normal protophloem differentiation pattern (Fig. 5A). Because inhibition of intracellular trafficking towards the PM by BFA treatment was required to observe elevated PtdIns(4,5)P_2_ levels upon *HsPIP5K* induction by its own biosensor (Fig. 5B), we speculate that PtdIns(4,5)P_2_ turnover at the PM might be too fast to be detected by microscopy techniques in normal conditions. However, elevated PtdIns(4,5)P_2_ cannot be observed by its biosensor in protophloem cells of *cvp2 cvl1* mutants (Fig. S4B), suggesting existence of other 5′ phosphatases or native PtdIns(4,5)P_2_ interactors that have higher affinity to the lipid than the biosensor itself. Overall, our results imply that an optimal degradation rate of PtdIns(4,5)P_2_ at the PM is required to prevent enhanced intracellular trafficking towards the vacuole, which would hamper protophloem differentiation and, in turn, its functionality.

## DISCUSSION

Vascular cell differentiation in *Arabidopsis* is a complex developmental process that involves the reinforcement of cell walls and total or partial cell clearance. Although tight PtdIns(4,5)P_2_/PtdIns4P homeostasis has been postulated to drive protophloem differentiation programs (Rodriguez-Villalon et al., 2015), little is actually known about the potential role of these lipids in the regulation of the vacuolar PCD associated with xylem differentiation (Gujas and Rodriguez-Villalon, 2016). By generating an estradiol-dependent genetic tool, we show that increasing PtdIns(4,5)P_2_ levels at the PM enhances intracellular trafficking towards the vacuole creating opposite developmental outcomes in terms of vascular differentiation (Fig. 2). This subcellular effect is directly responsible for the appearance of undifferentiated protophloem gap cells, the arrest of meristematic activity and cell growth observed in *cvp2 cvl1* or *pip5k1 pip5k2* double mutants (Fig. 1D-I, Fig. 4A) (Ischebeck et al., 2013; Rodriguez-Villalon et al., 2015). As defects in cell wall formation prevents an optimal vacuole-driven cell expansion, the reduced cell elongation rate observed upon boosting PtdIns(4,5)P_2_ levels might be partially due to interference with the trafficking of cellulose biosynthetic enzymes such as CESA6 (Fig. 2) (Fagard et al., 2000; Ugalde et al., 2016).

Intracellular trafficking and homotypic membrane fusion steps are crucial in vacuole biogenesis during xylem differentiation (Zhang et al., 2014). Enhanced trafficking to the vacuolar compartments positively correlates with the premature cell death and SCW formation observed in xylem cells upon *HsPIP5K* induction (Fig. 3B-G). Accordingly, the opposite phenotype was shown in *pip5k1 pip5k2* epidermal root cells (Ugalde et al., 2016), indicating that the PtdIns(4,5)P_2_-mediated intracellular trafficking shapes vacuolar morphology and, in turn, the time and rate of xylem differentiation (Fig. 3B-G). Elevated PtdIns(4,5)P_2_ levels accelerate vacuolar dynamics and PCD execution regardless of SCW dynamics (Fig. 3D-G), even if at this stage we cannot exclude the possibility that the reduced elongation rate observed in phosphoinositide and *cesa6*-deficient mutants might have an impact on PCD progression (Fig. 2F). Overall, our findings suggest that although vacuolar biogenesis, SCW formation and PCD must be coordinated during xylem differentiation, they require independent signals to be triggered. Notably, a recent study demonstrated that auxin – a pivotal regulator of xylem differentiation – requires intact phosphoinositide metabolism to limit vacuolar morphology and cell growth (Lofke et al., 2015). Several studies have also shown a feedback regulatory loop between auxin and phosphoinositide biosynthesis in root cells (Tejos et al., 2014). Hence, it is tempting to speculate that auxin modulates PtdIns(4,5)P_2_ concentration to regulate cellular expansion by modulating vacuolar dynamics.

Intriguingly, blocking vesicular recycling and trafficking towards the vacuolar compartment by BFA treatment triggered the opposite phenotypes in protophloem and protoxylem cells. Whereas in protoxylem cells BFA-mediated inhibition of trafficking from TGN to the vacuole seems to decrease the acuolar biogenesis rate required to sustain optimal xylem differentiation, protophloem elements of BFA-treated roots show a gradual fusion of late endosomes to the tonoplast, a phenotype reminiscent of that observed in *cvp2 cvl1* (Fig. 4F,G). BFA targets several ARF-GAP proteins including *VAN3/SCARFACE*, activity of which is stimulated by CVP2-derived PtdIns4P and is required for PIN1 recycling (Naramoto et al., 2009). Detailed examination of *sfc* loss-of-function mutants revealed the appearance of discontinuous protophloem strands after the transition zone (Fig. S4C). However, we cannot exclude at this stage the participation of other ARF-GAP factors involved in the PtdIns(4,5)P_2_-dependent regulation of cellular trafficking associated with protophloem differentiation. Nevertheless, our findings suggest the existence of specific protophloem master regulators whose functionality and/or localization are subjected to PtdIns(4,5)P_2_-dependent post-Golgi trafficking. This notion was reinforced by partially restoring *cvp2 cvl1* and *HsPIP5K*-triggered protophloem defects when blocking intracellular trafficking to the vacuole with WM treatments (Fig. 4I,J, Fig. S3D,E). Exogenous application of WM led to the disintegration of the atypical persistent vacuoles observed in mature units but it also restored cell wall morphology in the gap cells (Fig. 4I,J, Fig. S3D,E). Furthermore, formation of vacuolar structures upon BFA treatments precedes the appearance of gap cells (Fig. 4F), suggesting the interconnection of both processes. However, further experiments are required to assess whether the appearance of gap cells is a sign of a hampered protophloem differentiation process or an incomplete case of a premature differentiation program. Protophloem differentiated units, unlike mature xylem cells, are devoid of the appropriate subcellular machinery to degrade vacuolar compartments. Thus, it seems plausible that tight phosphoinositide levels at the PM/TGN act as a checkpoint for redirecting vesicular trafficking to the vacuole, implying the existence of a tissue-specific mechanism to control PtdIns(4,5)P_2_ turnover in protophloem cells. Although our findings demonstrate that CVP2 cycling to the PM in protophloem differentiating cells (Fig. 5A, Fig. S4A) contributes to modulate this process most likely by degrading PtdIns(4,5)P_2_ at the PM, we cannot exclude the existence of additional factors involved in the regulation of this process. Future investigations will decipher whether the gap phenotype observed in other protophloem mutants such as *octopus* or *brevis radix* (Rodriguez-Villalon et al., 2014) might be related to disturbed intracellular trafficking or other subcellular processes.

Overall, our data extends the current view of vascular cell differentiation, indicating that PtdIns(4,5)P_2_ and/or its downstream effectors coordinate these processes by modulating intracellular trafficking and vacuolar morphogenesis. However, further studies are required to identify the protophloem- and protoxylem-specific PM proteins internalized by the PtdIns(4,5)P_2_-dependent endocytic pathway that confer specificity to both vascular differentiation programs.

## MATERIALS AND METHODS

Molecular biology experiments such as plant transformation, genotyping or sequencing were performed as previously described (Rodriguez-Villalon et al., 2015).

### Plant material and growth conditions

All *Arabidopsis* transgenic lines were generated in the Columbia-0 wild-type background. The *cvp2 cvl1* and *sfc4* mutant alleles used in this study were previously described elsewhere (Carland and Nelson, 2009) as well as *UBQ::RabG3f-YFP*, *UBQ::VAMP711-YFP* and *UBQ::RabE1d-YFP* (Geldner et al., 2009). Phosphoinositide biosensors *UBQ::PH^FAPP1^-CITRINE* and *UBQ::PH^PLC^-CITRINE* were kindly provided by Dr Joop Vermeer and previously described (van Leeuwen et al., 2007; Vermeer et al., 2009). *PASPA3::ToIM* and *PASPA3::H2A-GFP* seeds were kindly provided by Dr Moritz Nowack (VIB-University of Ghent, Belgium) whereas *CESA6::YFP-CESA6*, *CESA6::TDT-CESA6*, *IRX3::IRX3-YFP* and *prc1-1* seeds were generously provided by Dr Clara Sanchez-Rodriguez (ETH-Zurich, Switzerland). Tissue-specific reporter lines were either obtained from Dr Niko Geldner (University of Lausanne, Switzerland) (*CASP1::CASP1-GFP*) or directly from the Nottingham *Arabidopsis* stock center (*S18::GFP*). Seeds were stratified in the dark at 4°C for 48 h and grown on vertically orientated ½ Murashige and Skoog (MS) medium plates under continuous light conditions. Treatments were performed on 4-day-old seedlings that were grown on ½ MS plates and then transferred to plates supplemented with the indicated amounts of estradiol, BFA and WM. All chemical compounds were obtained from Sigma except FM4-64, which was purchased from Thermo Fisher Scientific. Each drug was dissolved in DMSO (estradiol, BFA and WM) or water (FM4-64, DAPI) and then diluted in autoclaved culture media.

### DNA construct preparation

Gateway cloning was used to obtain the *UBQ::XVE::HsPIP5K* construct by recombining the pENTRY-HsPIP5*K* plasmid into the modified pMDC7 vector (Lee et al., 2013). In order to monitor *HsPIP5K* tissue-specific expression, 2 kb of genomic DNA region of *DMP4*, *AHP6* and *BAM3* promotor sequence were introduced using *Pme*I/*Bst*BI restriction digestion and ligated directly with pMDC7. All primer sequences are presented in Table S1. Additionally, a GFP-tagged *HsPIP5K* fragment was amplified after introducing the *HsPIP5K* construct into the GFP-harboring Gateway plasmid pK7wgf2 using the primers *eGFP_attB1_F* and *HsPIP5Kstop_attB2_R*. The final construct was introduced into pDONR207 (Invitrogen) and recombined into the above-mentioned estradiol-inducible vector. Additionally, *AHP6::NLS-3xVENUS* and *DMP4:NLS-3xVenus* were the result of introducing *AHP6* or *DMP4* promoters into pDONR P4-P1r vectors and their final recombination together with *pENzeo*-*NLS3xVENUS* into destination vector pEDO 097 (Vermeer et al., 2014). Finally, to generate the *CVP2::CVP2-CITRINE* construct, a plasmid containing 2 kb of the genomic region upstream of CVP2 (Rodriguez-Villalon et al., 2014) was recombined with a pENTRY vector containing the coding sequence of CVP2. The CVP2 coding sequence was amplified using the primers *CVP2cDNA_F* and *CVP2cDNA_R.* The above-mentioned plasmids were recombined together with a pENTRY-CITRINE into a pH7m34GW destination vector.

### Microscopy and phenotype analysis

For analyzing the root meristem size and cell elongation rate, 6-day-old seedlings were stained with 10 µg/ml propidium iodide (PI, Sigma) and imaged by confocal (Zeiss LSM 780 instrument) or multi-photon (Leica SP8 instrument) microscopy. Root cortical cells from at least 12 seedlings were counted from the quiescence center to the transition zone in order to estimate meristem size. To estimate the cell elongation rate, images were processed by ImageJ and cell length was measured from transition zone onwards. Means and standard error were calculated and statistical significance was evaluated by the Student's *t*-test. Representative images of each experiment are displayed, and images of the same panel were represented with comparable objective magnification and zoom. To analyze FM4-64 uptake, seedlings pre-treated with 1 µM estradiol for 5 h were incubated for 2 min in MS liquid media supplemented with 2 µM FM4-64. Afterwards, seedlings were transferred to new MS liquid media supplemented with estradiol or DMSO (mock) and analyzed by multi-photon confocal microscope at the above-mentioned time points. To assess meristematic activity and the size of the elongation zone, time-course experiments at 12, 24 and 48 h after *HsPIP5K* induction were performed in *UBQ::XVE::HsPIP5K*, *AHP6::XVE::HsPIP5K* and *DMP4::XVE::HsPIP5K* roots followed by Calcofluor White M2R dye (18909, Sigma) staining and confocal microscopy imaging. To visualize vascular organelles, roots were stained with PI and visualized using multi-photon confocal microscopy with a 40× objective of a Leica SP8 microscope. To estimate cellulose content, seedlings were first cleared and fixed using the ClearSee protocol (Kurihara et al., 2015) and then stained with Calcofluor White M2R dye. Roots were then mounted in ClearSee solution and visualized (405 nm excitation and 425-475 nm emission).

### Phosphoinositide measurements

^32^Pi uptake and incorporation into phosphoinositides in 6-day-old seedlings was measured as described (Munnik and Zarza, 2013). Three seedlings per sample were incubated for approximately 20 h with 10 µCi ^32^Pi orthophosphate prior to lipid extraction. Lipids were then separated by TLC, visualized by phosphoimaging on a Typhoon 8600 scanner, and quantified by QuantityOne software. PtdInsP and PtdInsP_2_ incorporation is the mean of three biological replicates.
